# Composite Eco-Friendly Sound Absorbing Materials Made of Recycled Textile Waste and Biopolymers †

**DOI:** 10.3390/ma12234020

**Published:** 2019-12-03

**Authors:** Chiara Rubino, Marilés Bonet Aracil, Jaime Gisbert-Payá, Stefania Liuzzi, Pietro Stefanizzi, Manuel Zamorano Cantó, Francesco Martellotta

**Affiliations:** 1 Dipartimento di Scienze dell’Ingegneria Civile e dell’Architettura, Politecnico di Bari, via Orabona 4, I-70125 Bari, Italy; chiara.rubino@poliba.it (C.R.); stefania.liuzzi@poliba.it (S.L.); pietro.stefanizzi@poliba.it (P.S.); 2 Grupo de Investigación en la Industria Textil (GIITEX), Departamento de Ingeniería Textil y Papelera, Universitat Politècnica de València, 46022 Alcoy, Alicante, Spain; maboar@txp.upv.es (M.B.A.); jaigispa@txp.upv.es (J.G.-P.); mzamoran@upvnet.upv.es (M.Z.C.)

**Keywords:** textile waste, biopolymers, sound absorption, sustainable materials, circular economy

## Abstract

In recent years, the interest in reusing recycled fibers as building materials has been growing as a consequence of their ability to reduce the production of waste and the use of virgin resources, taking advantage of the potential that fibrous materials may offer to improve thermal and acoustic comfort. Composite panels, made of 100% wool waste fibers and bound by means of either a chitosan solution and a gum Arabic solution, were tested and characterized in terms of acoustic and non-acoustic properties. Samples with a 5 cm thickness and different density values were made to investigate the influence of flow resistivity on the final performance. Experimental results demonstrated that the samples had thermal conductivity ranging between 0.049 and 0.060 W/(m K), well comparable to conventional building materials. Similarly, acoustic results were very promising, showing absorption coefficients that, for the given thickness, were generally higher than 0.5 from 500 Hz on, and higher than 0.9 from 1 kHz on. Finally, the effects of the non-acoustic properties and of the air gap behind the samples on the acoustic behavior were also analyzed, proving that the agreement with absorption values predicted by empirical models was also very good.

## 1. Introduction

Materials used in the construction industry for thermal insulation and noise control are mainly inorganic and synthetic composites, i.e., glass wool, stone wool, and polystyrene. These materials, despite having low thermal conductivity values and high sound absorption coefficients, cause significant environmental impacts during their production processes [1] and may affect human health depending on fiber dose, dimension, and durability [2]. Conversely, one of the greatest challenges for future buildings is to guarantee low energy use, preserving indoor thermal and acoustic comfort by using bio-based components able to ensure a healthy and sustainable environment. Choosing non-toxic, environment friendly, and recyclable building composites implies a growing attention in testing natural (vegetal/animal) or waste fibers as an alternative to mineral and synthetic ones [1].

Many researchers have been carrying out studies about innovative “green” building materials. The difficult disposal of agro-residues has stimulated the interest of the research community in the possible use of agricultural waste products as fibrous matrix of bio-based composites [3]. Furthermore, the wide availability of many “local” natural materials encouraged several researchers to study their sound absorption and thermal insulation properties. Thus, olive tree leaves [4], flax, hemp [5], cotton stalk [6], straw bales [7], kenaf fibers [8], coir fibers [9], rice straw [10], and others [11] have been considered.

The disposal issue concerns agricultural by-products, as well as the apparel industry, which is responsible of an enormous volume of wastes resulting from a fast-changing fashion culture based on designing garments characterized by an intrinsic obsolescence. A textile fabric can provoke pollution from the earliest manufacturing process (pre-consumer waste) to the end of its useful life (post-consumer waste) [12]. Thus, the search for a proper way to reuse textile fibers is stimulating several attempts to use them in the construction industry, especially in the form of sustainable panels [13,14] or mats [15]. The reuse of waste or by-products as new raw materials for innovative and sustainable building components has the important advantage of creating a “circular economy” system which transforms discarded fibers into useful goods with added value. In addition, ecological benefits related to a lesser use of virgin resources and to the limited need for landfill are achieved [16].

Manufacturing a sustainable building material means controlling the type and amount of energy used for its production, as well as verifying specific requirements on the possibility to recycle or reuse the material at the end of its useful life and on the toxicity of all its components, including the binder together with raw materials [1,2]. 

In terms of environmental impact of wool recycling, any quantitative assessment is very difficult and affected by high uncertainty, mostly because wool as a virgin material is a co-product with meat. Thus, depending on what we assume to be the main product, the climate impact (estimated by the global warming potential (GWP)) of wool fibers may range from 36.2 kg CO_2_ equivalents per kg fibers, to 26 kg CO_2_ equivalents per kg fibers [17]. Geographic factors may also significantly affect the above results. By assuming the process of mechanical unravelling of wool to low grade wool yarns to use as a substitute of polyester fibers in blankets, it has been calculated that a significantly positive effect can be obtained [18], compared to incinerating or sending to landfill the wool waste. In particular, in the mentioned study the recycling process assumes that wool may be carded and spun into new yarn to replace other fibers and, consequently, avoiding the largest impact due to the fact that raising of sheep alone accounts for more than 50% of the climate change impacts in the life cycle of wool and about a third of the energy consumption. 

With reference to binders, despite the increasing popularity of chitosan in the literature, only one reference [19] could be found discussing its life cycle assessment (cradle to gate). Results showed impressive differences depending on the supply chain, with a climate change impact of 77 kg CO_2_ equivalent per kg of chitosan for the European market, and 12.2 kg CO_2_ equivalent per kg of chitosan for the Indian supply chain. 

Gum Arabic, despite its much older history, and widespread use in the food and drug industry has, to the best of the authors knowledge, never been investigated in terms of environmental impact and life cycle assessment. However, gum Arabic is derived from Acacia plants that grow in fairly dry climates (sub-Saharan Africa and West Asia), and it also contributes to fertilize soil by fixing nitrogen, contrasting desertification. None of the manufacturing processes usually require thermal energy, with transportation and mechanical breaking likely having the largest share of the environmental impact. 

In the study reported in this paper, 100% recycled wool composite panels are designed as an alternative to conventional solutions aiming at a sound absorption coefficient of at least 0.5 from 500 Hz on, and a thermal conductivity of at least 0.05 W/(m K). The tested samples were made by using two different natural binders based on chitosan and gum Arabic. Chitosan is a polysaccharide obtained from alkaline deacetylation of chitin from crustacean shells and, thanks to its commercial availability, it is particularly used as a film and adhesive [20]. Several researches [21,22] have been carried out in order to investigate its binding properties. Gum Arabic is a commercially available dried gummy derived from the exudation of steams and branches of Acacia trees. Thanks to its physical and mechanical properties, gum Arabic can be considered a valid substitute for formaldehyde or any other conventional binder in the building materials field [23]. Furthermore, it may be added to cements to improve concrete mechanical properties [24]. The use of organic adhesive solutions to bond recycled post-consumers wool waste allows to produce recyclable materials without further impacts on the environment. The high nitrogen content makes natural fibers biodegradable in a few years unlike synthetic ones [25].

The aim of the research is to characterize the sound absorbing behavior (and other related properties such as thermal insulation) of the innovative tested materials by taking into account, in particular, the effect played by density and porosity variation. The paper also focuses on the study of the suitable placement of the proposed materials by analyzing variation in sound absorption coefficients as a function of the distance from the backing wall. Finally, in order to obtain further elements contributing to material optimization, all the experimental acoustic results were compared with theoretical results based on the empirical prediction model proposed by Delany and Bazley (D&B) [26] and on the phenomenological model suggested by Johnson, Champoux, and Allard (JCA) [27,28].

## 2. Materials

### 2.1. Basic Components

Two different binders were used, one from vegetable resources (gum Arabic) and the other one from animal ones (chitosan). Gum Arabic is a hardened sap from the Acacia tree and is commonly used as a natural gum. Its chemical composition is a complex polysaccharide with high molecular weight, water soluble, and its solution gives a slight yellow to reddish color. Gum Arabic is considered a biopolymer. It was purchased from Lana y Telar from Spain. 

On the other side, chitosan is also a polysaccharide from crustacean by-products. It is considered a biopolymer and is comprised of partially deacetylated units of 1–4, D-glucosamine. It was purchased from chitosan of medium molecular weight from Sigma-Aldrich. Chitosan is capable of crosslinking itself or by means of a crosslinking agent. In this occasion no crosslinking agent was included. Acetic acid to solubilize chitosan was supplied by Panreac.

Wool is a natural fiber obtained from animals and it is mainly characterized by its proteinic chemical structure and the thermal insulation. It is the natural fiber with the highest limit oxygen index (LOI) which confers resistance against fire ignition. Merino wool fibers used in this study were derived from discarded shreds resulting from the manufacturing process of an Italian clothing company (Gordon Confezioni, Bari, Italy).

### 2.2. Sample Preparation

**Figure 1:**
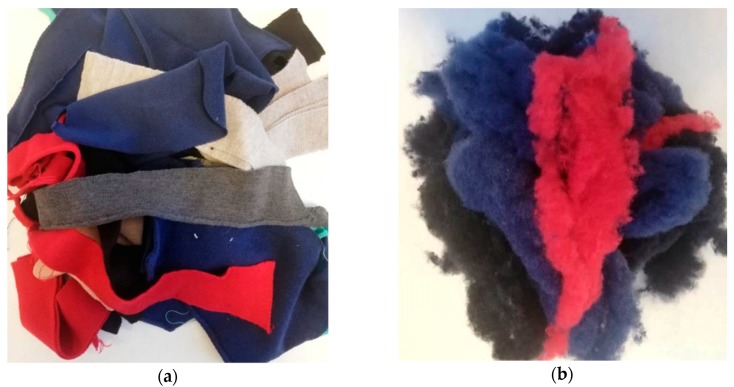
Textile waste in the form of cut fabrics (**a**) and fleece matrix used as raw material (**b**).

The experimented materials were prepared by using 100% Merino wool fibers initially available in the form of cut fabrics (Figure 1a); then, a soft wool batting (Figure 1b) was obtained by carding and by scouring the fibers.

As anticipated, chitosan and gum Arabic were chosen as the natural binders to investigate. Two solutions were achieved by dissolving different amounts of chitosan and gum Arabic in water, as outlined in Table 1. Both blends were mixed in a magnetic stirrer, at room temperature and relative humidity, for about 1 h. The concentration of solutes was chosen, after several attempts, in order to obtain the best combination of porosity and compactness. Finally, materials with potential use as mats with good thermal and acoustic performance were produced.

Two different groups of final composites were produced: The first one was based on textile waste bound with chitosan solution (subsequently referred to as CH); the second type was prepared by binding textile waste with the gum Arabic solution (subsequently referred to as GA).

A three-step process was used to produce the samples. First, the fibers were transformed into wool batting. The second step consisted of soaking the raw materials in the binding solution to let the wool get fully impregnated. The excess amount of liquid was removed by squeezing, mostly to avoid the exceeding binder, that once solid, might compromise a homogeneous distribution of porosity in the final specimen (e.g., by creating surface crusts). In the third step, the achieved mix was compressed into PVC molds to form cylindrical samples. Finally, the samples were dried in an oven at 100 °C, for 1 h and, after drying, they were left in desiccators containing silica gel, under controlled conditions to reach their mass stabilization before testing.

**Figure 2:**
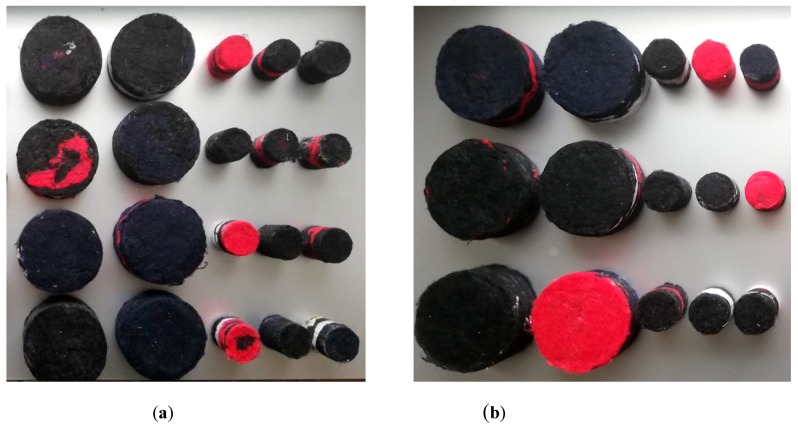
Samples with chitosan binder solution (**a**) and samples with gum Arabic binder solution (**b**).

Several mixes with different bulk densities and same percentages of binder and fibrous matrix were prepared (Table 2). Binder concentration was expressed both as a percentage in wet mass (obtained by weighing the fibers before soaking and after removing the excess binder), and in dry mass (obtained by weighing the final products, after drying them in the oven). Bulk density values are given as mean values among six measurements [29] given that, for each type of binder and for each density value, six cylindrical specimens, 5 cm thick, were prepared (Figure 2). Three were prepared in 10 cm diameter to measure thermal conductivity, thermal diffusivity, specific heat capacity, and normal incidence absorption coefficient at medium-low frequencies. The remaining three samples were prepared in 4 cm diameter to measure normal incidence absorption coefficient at high frequencies and non-acoustic properties (air flow resistance, porosity, and tortuosity).

### 2.3. Microstructural Analysis 

A scanning electron microscopy (SEM) analysis was carried out using a Fei Phenom desktop scanner (Thermo Fisher Scientific, Hillsboro, OR, USA). In order to compare the effect of the binder addition on the fibers, samples with and without the binding solution were tested. The samples were prepared by cutting small slices of the fibrous composites onto an adhesive carbon tab attached to an aluminum stub. A sputter coating in gold for a period t = 15 min was applied.

**Figure 3:**
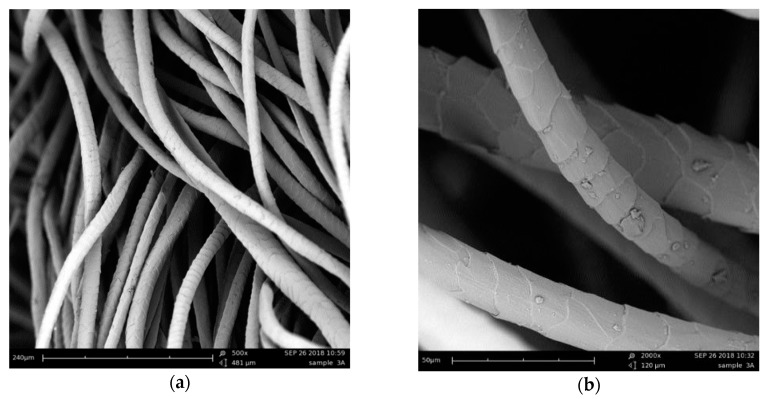
Cuticular scale pattern of wool fibers at 500× (**a**) and 2000× (**b**).

Wool is an animal fiber with keratin as the main protein component (42% in volume). It exhibits a complex and sophisticated structure with the outermost layer (i.e., cuticle) overlapping by scaly cells which are about 10% (in volume) of the fiber. These scales played an important role in the manufacturing process of the building components simplifying the fibers intertwining. During repeated mixing and pressing actions, the scales edges bended, became interlocked and entangled enabling a good cohesion of the resulting fibrous matrix [30]. Figure 3 shows the SEM images of wool fibers without any binder, in which the scale patterns of the cuticle can be observed. According to Patnaik et al. [31], these scales were also effective in dampening the sound wave, especially in the medium high frequency range. 

**Figure 4:**
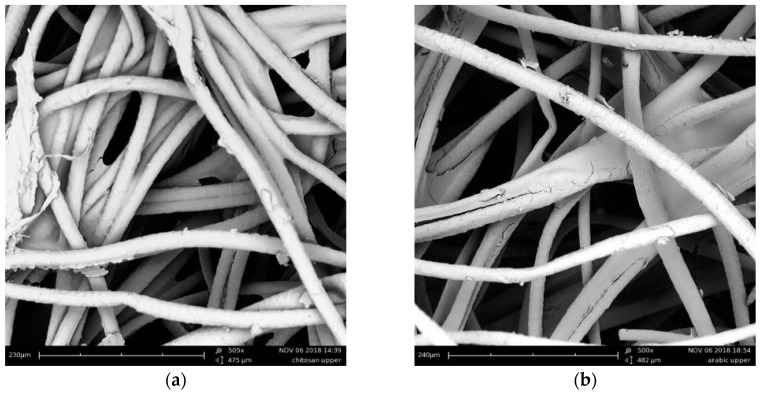
Wool samples with chitosan binder (**a**) and gum Arabic binder (**b**) at 500×.

Figure 4 shows the SEM images of the fibers bonded with chitosan and gum Arabic solutions. The resulting composite materials were characterized by cylindrical fibers with a 20 μm average diameter, distributed more or less randomly in plane. Both binding solutions joined the fibers preserving the porous matrix of the final composite materials. The fibers intertwined creating a network of tiny air pockets which guaranteed a highly porous product with potential good thermal and acoustic performances.

## 3. Methods

### 3.1. Basic Physical Properties

A ULTRAPYC 1200-e Quantachrome Helium gas pycnometer was used to measure the true density *ρ*_true_ of the samples. Subsequently, open porosity (*ε*) was evaluated from the obtained true density and from the bulk density *ρ*_bulk_, previously measured according to the gravimetric method. The open porosity of textile fibrous materials corresponds to the inter-fiber and intra-fiber porosity.

*ε* = 1 − *ρ*_bulk_/*ρ*_true_ (1)

Tortuosity (*τ*) was measured in order to evaluate the influence of the pore micro-geometry (i.e., the distribution, the size, and the shape of the pores) on the sound propagation path length in the materials under investigation. As they are non-conducting porous absorbers, the method proposed by Brown [32], based on an electro-acoustic analogy was chosen. According to the authors of [33], three samples for each mix were saturated by soaking in a 10% copper sulfate solution (CuSO_4_) and, after 24 h, a current was applied using two circular copper plates as electrodes. The electrical resistivity R_0_ was calculated for each sample after recording the current intensity values, by varying the voltage between 1 and 8 V. The electrical resistivity of the conducting fluid R_W_ was also obtained. The tortuosity was given by the following Equation:

*τ* = *ε* (*R*_0_/*R_W_*) (2)

where *ε* is the porosity of the sample.

The tortuosity was also empirically estimated according to Berryman’s formula [34]:

*τ_est_* = 1 + (1 − *ε*)/2*ε* (3)

Air flow resistance (*σ*_s_) of three samples from each mix was measured according to the method proposed by Ingard and Dear [35] which uses sound waves. Although this procedure could not be considered as steady as the standardized approach, del Rey et al. [36] proved that it was scientifically accurate when adopted for thin or scarcely resistive samples, as occurs in the present research. 

The measurement setup consisted of a 5 mm thick methacrylate tube, with a 4 cm inner diameter. The tube was divided in two parts; each of them 85 cm long. At one end there was a 5 cm loudspeaker (Visaton FRS 5, Visaton, Haan, Germany) with a frequency response spanning from 150 to 20 kHz; at the other end there was a rigid termination made by a 5 cm thick methacrylate. The tested samples were assembled between the two tubes. The flow resistance was deduced using the transfer function of the two microphones excited by an exponential sine sweep. All of the processing was performed by a custom-made MATLAB^®^ (2018, Mathworks, Natick, MA, USA) graphic user interface.

Thermal performances were measured, on three samples for each typology, using a transient plane source apparatus, ISOMET 2104 (Applied Precision, Bratislava, Slovakia). This device was used by several authors [37,38] in order to test the thermal behavior of fibrous materials. The measurement was based on the analysis of temperature response of each sample to heat flow impulses [39]. Prior to testing the thermal properties, all the specimens were oven dried at 105 °C then cooled to ambient temperature in desiccators containing silica gel. The dry-state thermal conductivity *λ*_dry_ was then measured. 

### 3.2. Acoustic Properties

According to ISO 10534-2:1998 [40], sound absorption measurements were performed by the transfer function method. Two tubes with different diameters (10 and 4 cm) and a thickness of 5 mm were used for the test with the aim to consider the largest spectrum range. The tube with an internal diameter of 10 cm had a maximum measurable frequency of 2 kHz and it used two different microphone distances (6 and 20 cm, respectively yielding a low frequency limit of 400 and 50 Hz). The emitting end consisted of an 11 cm loudspeaker sealed into a wooden case and suitably isolated from the tube structure by an elastic and protective layer. The second tube, with a diameter of 4 cm, was the same used for the flow resistance measurement. In this case the microphone spacing was 3 cm, and the frequency covered a range between 200 and 5 kHz. All the processing was performed by a MATLAB^®^ (2018, Mathworks, Natick, MA, USA) graphic user interface generating a 5 s linear sweep from 70 to 3 kHz, used in combination with the largest tube, and from 500 to 5 kHz considering the smallest tube. 

The measured sound absorption coefficients were compared with those estimated by theoretical prediction models. The empirical model proposed by Delany and Bazley [26] and the phenomenological model developed by Johnson, Champoux, and Allard [27,28] were chosen. In both situations, the absorption coefficients of the investigated fibrous materials were obtained after determining the characteristic impedance *Z*c and the wavenumber *k*. The empirical model just links the sound absorption coefficients to the air flow resistivity *σ* (i.e., the air flow resistance per unit thickness) of the materials. Thus, to solve the D&B equations only one non-acoustic property was used to calculate the parameter *X* = (*ρ*_0_ *f*)/*σ* (*f* being the frequency and *ρ*_0_ being the density of the air), needed to determine the characteristic impedance *Z*_C_ and the wavenumber *k*:

*Z*_C_ = *ρ*_0_*c*_0_ (1 + 0.057*X*^−0.754^ − *j*0.087*X*^−0.732^) (4)

*k* = (*ω*/*c*_0_) (1 + 0.0978 *X*^−0.700^ − *j*0.189*X*^−0.595^) (5)

where *c*_0_ is the speed of sound in air and *ω* is angular frequency. The D&B equations can be considered valid only in a defined frequency range given by 0.01 < *X* < 1.0, and for *σ* values below 50 kN∙s/m^4^ [41].

Although the D&B empirical model has been successfully tested over a variety of fibrous materials having porosity close to unity (similar to those under investigation), it nonetheless neglects the important effects that other structural parameters of the materials may have to influence their acoustic performance. For this reason, the JCA model was also considered, according to which *Z*_C_ and *k* are expressed as a function of the effective (or dynamic) bulk density *ρ*_e_ and bulk modulus *k*_e_:

*Z*_C_ = (*k*_e_*ρ*_e_)^0.5^ (6)

*k* = (*ρ*_e_/*k*_e_)^0.5^ (7)

The computation of these two parameters involves the use of other physical quantities including porosity *ε*, tortuosity *τ*, viscous characteristic length *Λ,* and thermal characteristic length *Λ′*, in addition to flow resistivity and density. Most of such coefficients (porosity, tortuosity, flow resistivity, and density) were analyzed in Section 3.1. A more extensive explanation of the physical meaning of the two characteristic lengths is beyond the scope of the paper, so reference to the original paper [28] or textbooks [41] is suggested. Briefly, the two characteristic lengths describe the effects of viscosity and thermal dissipative forces inside the porous structure. The viscous characteristic length is the weighted ratio of the volume to surface area of the pores. It takes into account some significant parameters, i.e., the pore shape represented by a variable *s* which reflects the deviation of pore shape from an ideal circle and lies between 0.3 and 3. The thermal characteristic length is needed for materials showing a complex internal structure. Usually, *Λ′* ≥ *Λ* and, in first approximation, *Λ′* = 2 *Λ*.

### 3.3. Ignitability Test

An ignitability test was carried out, as far as possible according to ISO 11925-2:2010 [42]. The major non-standard compliant element was represented by the shape and dimension of the sample that, for this batch, was only produced in cylindrical molds having 10 cm diameter and 5 cm thickness. Thus, an “edge exposed” test was carried out, with a 15 s flame application, on two different samples of the selected typology. A reference sample, made of pressed wool fibers (3 cm thick) was used as a reference for comparison purposes, together with GA-3 and CH-4 samples.

## 4. Results

### 4.1. Basic Physical Properties

The results of the non-acoustic parameters are given in Table 3. The values of porosity, tortuosity, air flow resistance, and air flow resistivity are given as mean values of the experimental data together with their measurement uncertainty calculated according to Reference [29]. Air flow resistivity (i.e., the air flow resistance per unit thickness) was also calculated as it is more frequently used as a criterion for choosing suitable materials for noise control applications. As seen in Section 3.2, it also plays an important role as an input parameter in prediction models.

As shown in Table 3, all materials were characterized by a porosity close to 0.9, regardless of the nature of the binder. This was in agreement with the porosity values of felts and mineral fiber materials ranging respectively from 0.83 to 0.95 and from 0.92 to 0.99 [43]. A void fraction close to 90% was expected in view of the microstructural analysis carried out in Section 2.3.

Tortuosity could not be measured for GA samples because gum Arabic is soluble in water and this would have prevented a suitable placement of the samples in the measurement equipment. Thus, in this case *τ* was only estimated according to Equation (3). A comparison between measured and predicted values, both available for CH samples, showed that predicted values were slightly underestimated with differences between 5% and a maximum of 15%. Thus, similar differences might be expected in the GA samples. In both cases, tortuosity values are in agreement with close-to-unity values typical of fibrous materials, where the fluid always takes the straightest path due to the absence of solid grains [44]. Considering that tortuosity may assume much larger values (e.g., up to 4 for densely packed granular materials), a 10% discrepancy may be considered negligible and, more importantly, of no significant effect on the sound absorption coefficients (as shown in next section). 

**Figure 5:**
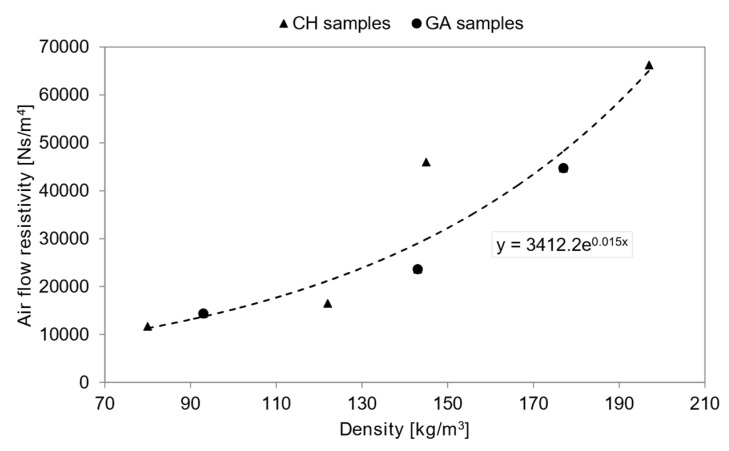
Experimental air flow resistivity versus density.

As confirmed by results reported in Table 3, air flow resistivity for all tested materials was below 100 kN∙s/m^4^, which is the limiting value for a material to be considered as an impervious layer, as reported by del Rey et al. [45], and only in one case it exceeded 50 kN∙s/m^4^ corresponding to the limiting value for the application of D&B model. For all samples air flow resistivity proved to be well correlated to bulk density values (Figure 5), as demonstrated by the statistically significant (R^2^ = 0.8891) exponential correlation that was found.

The experimental results obtained for non-acoustic properties of the studied materials were in agreement with some vegetal woolen building materials with similar density values [46,47]. Glé et al. [46] carried out a research about the acoustic behavior of vegetal woolen materials and showed that these biobased panels had high porosity and a tortuosity of about one, similar to more conventional fibrous composites. Additionally, an increase of the air flow resistivity values with the increase in density was observed. A similar dependence on density was found for the effective thermal conductivity. It can be noted that both CH and GA samples showed similar thermal conductivity values as a function of the bulk density (Table 3). CH samples had λ varying between 0.049 and 0.060 W/(m K) with a bulk density *ρ* ranging from 80 to 197 kg/m^3^, while for GA samples, *λ* varied from 0.050 to 0.059 W/(m K) with bulk density *ρ* ranging between 93 and 177 kg/m^3^.

**Figure 6:**
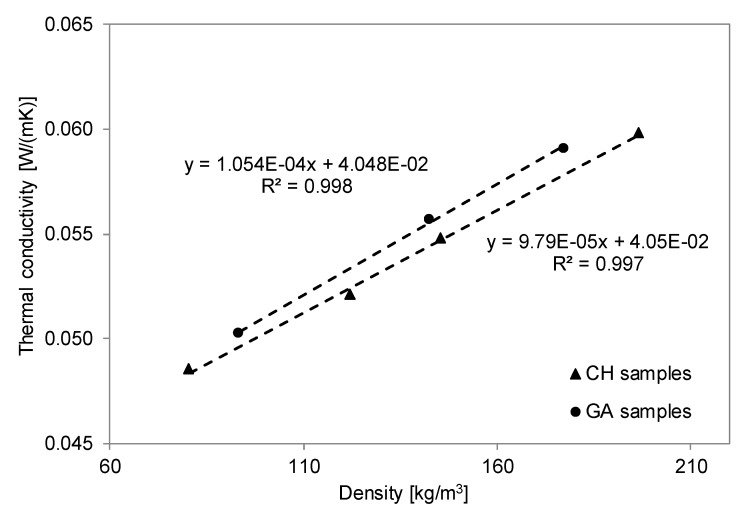
Thermal conductivity λ_dry_ versus bulk density ρ for chitosan (CH) and gum Arabic (GA) samples.

Figure 6 shows the thermal conductivity values of CH and GA samples as a function of their density values. Statistically significant linear correlations were found for both samples (R^2^ = 0.997 for CH and R^2^ = 0.999 for GA). Similar linear trends were observed for the two groups of materials, with λ decreasing linearly when the density dropped. However, a statistically significant difference between the two regressions was found (with a residual probability *p* < 0.005), resulting in CH samples having a thermal conductivity about 7% lower than a same-density GA sample. This small difference does not seem to be related to the sample porosity, which was very similar for same-density samples, neither could be better explained by the analysis of the microstructure. In fact, the SEM images (Figure 3) demonstrated that the use of different binders barely affected the porous matrix of the final composite (Table 3). Conversely, the different fraction of binder (on the dry sample) outlined in Table 2, may well explain the 7% variation in thermal conductivity. In fact, GA samples in which the same amount of wool fibers is bound by a 22% in mass of binder, against the 5% of CH samples. 

### 4.2. Acoustic Properties

The characterization of the acoustic performance was carried out by comparing the normal incidence absorption coefficients measured in the laboratory with those predicted using Delany–Bazley and Johnson–Champoux–Allard equations. Data used to feed the D&B model, i.e., air flow resistivity, were derived from experimental measurements (Table 3). The calculation of JCA predictions was carried out using some previously measured parameters, i.e., density, porosity, tortuosity, and air flow resistivity (Table 3), while the values for the shape factor and the characteristic length ratio *Λ′*/*Λ* were numerically adjusted to get the best agreement between measured and predicted values. The resulting set of parameters is given in Table 4. A comparison between results obtained using measured and estimated tortuosity confirmed that differences in the relevant mean absolute errors were usually negligible, thus only estimated values were used in all the cases. 

**Figure 7:**
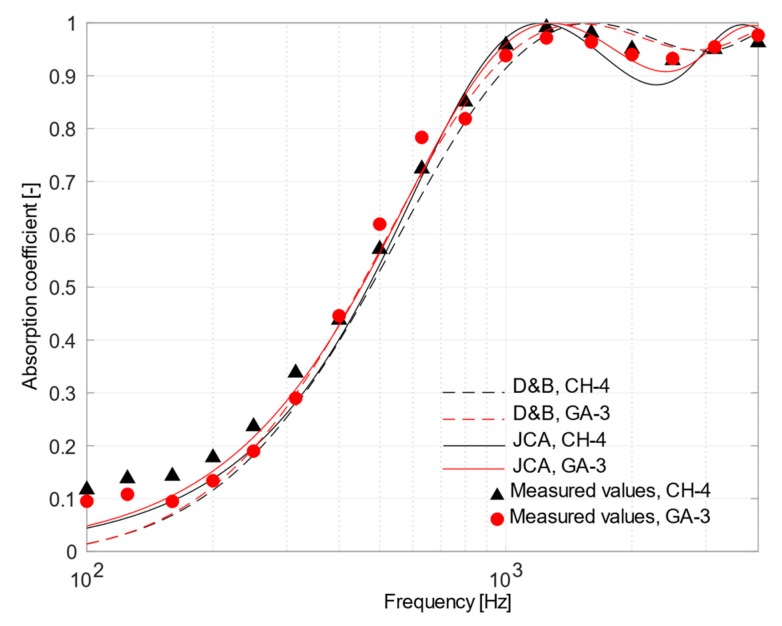
Plot of mean measured normal incidence absorption coefficients and those predicted using Delany and Bazley and Johnson–Champoux–Allard equation for CH-4 sample (in black) and GA-3 sample (in red).

Figure 7 shows the normal incidence sound absorption for CH-4 and GA-3 samples, both having low and comparable air flow resistance values. As it can be observed, the experimental curves of the two materials were almost overlapping and both trends were in accordance with those estimated by the theoretical approaches. In particular, the JCA model allowed a rather precise estimation of peak placement, its maximum–minimum fluctuations and values of absorption coefficients. The phenomenological model perfectly predicted the first peak appearing around 1250 Hz with α close to unity and the following drop around 2500 Hz. It is important to point out that optimization of the estimated parameters had a negligible effect on the final result.

**Figure 8:**
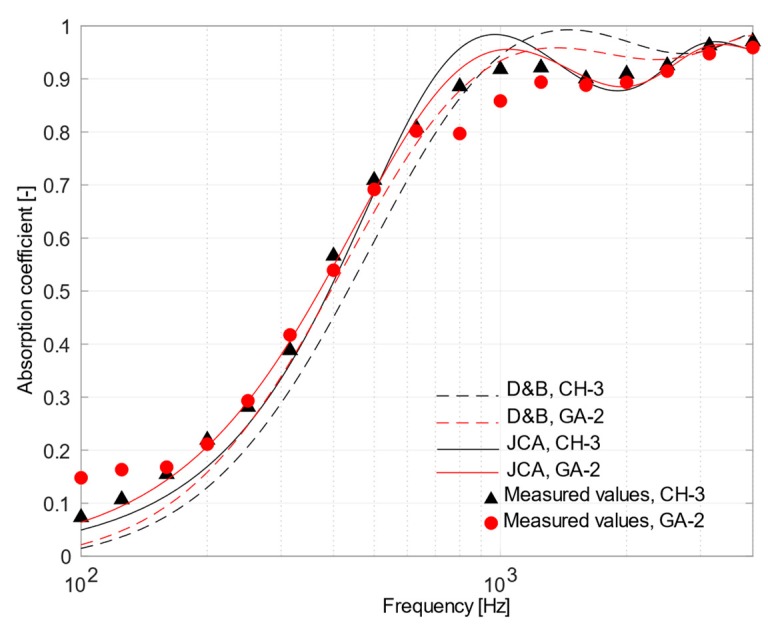
Plot of mean measured normal incidence absorption coefficients and those predicted using Delany and Bazley and Johnson–Champoux–Allard equation for CH-3 sample (in black) and GA-2 sample (in red).

Figure 8 shows a comparison between CH-3 and GA-2 samples. The curves of the two samples were similar up to 630 Hz, where some differences could be noted. For GA-2 a first mild peak appeared around 630 Hz, followed by another peak around 1250 Hz, almost coinciding with the first peak of CH-3 sample. The measured values showed good agreement from 1600 Hz on. Considering the CH-3 case, the phenomenological model performed better than the empirical one. The first peak predicted by JCA model, although overestimated, was almost aligned to the measured one. Taking into account the GA-2 material, significant discrepancies appeared between 600 and 1600 Hz where measured values were lower than expected according to the models, and apparently no combination of parameters could return a similar behavior. Thus, the discrepancy should suggest that some anomalous behavior took place with GA-2 samples.

A comparison between Figure 7 and Figure 8 pointed out that the use of different binders played a negligible role in changing the acoustic performance of the compared samples (CH-3, CH-4, GA-2, and GA-3), while the structure of the materials mainly affected their acoustic behavior. All the samples, apart from limited exceptions, showed sound absorption coefficients which were consistent with that of porous materials of the same thickness and having the corresponding flow resistance values (ranging between 585 and 1182 Ns/m^3^). However, the fact that JCA model showed a better agreement than D&B confirmed the importance of the microstructure in altering the sound path and, for example, moving the peak of absorption towards lower frequencies (compared to the expected theoretical peak which should appear at the frequency having the quarter-wavelength equal to the sample thickness). As shown in Table 4, the shape factor estimated for CH-3, CH-4, GA-2, and GA-3 samples was larger than one (ranged between 2 and 3), demonstrating that despite tortuosity being close to unity, the fibers are arranged according to an intricate internal structure, which is far from the “ideal” cylindrical pores.

**Figure 9:**
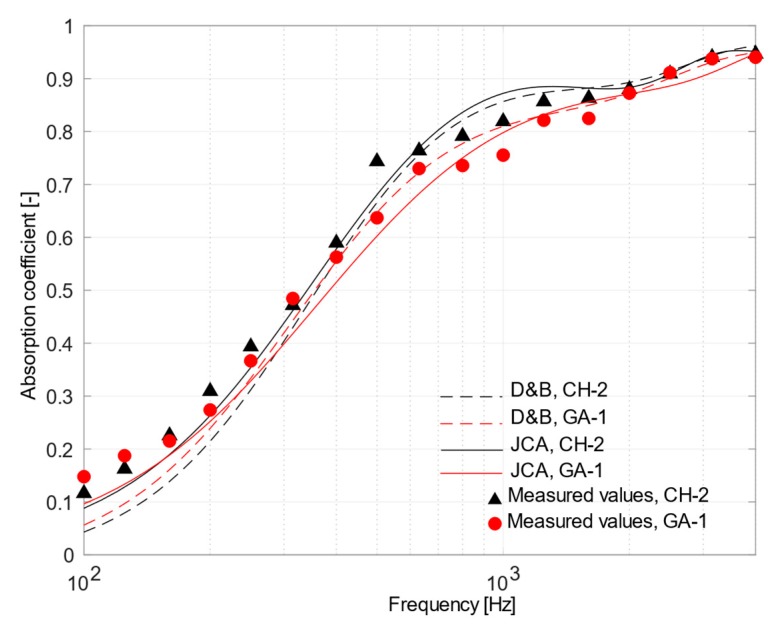
Plot of mean measured normal incidence absorption coefficients and those predicted using Delany and Bazley and Johnson–Champoux–Allard equation for CH-2 sample (in black) and GA-1 sample (in red).

Figure 9 shows the sound absorption curves for the samples with higher flow resistance (CH-2 and GA-1). Clearly, the absorption coefficients differed significantly from those previously analyzed, as they increased at low frequencies and decreased above 800 Hz. At 315 Hz, α was about 0.5 for GA-1 and CH-2 samples against a value of about 0.3 for CH-4 and GA-3 samples and 0.4 for CH-3 and GA-2 samples. At 1250 Hz, α was about 0.8 for GA-1 and CH-2 samples against a value of about 1 for CH-4 and GA-3 samples and 0.9 for CH-3 and GA-2 samples. This was because samples CH-2 and GA-1 had high air flow resistivity values (46 and 44.7 kN∙s/m^4^, respectively) that increased the viscous and thermal interaction inside the micro pores forming the material, while increasing, at the same time, the surface impedance.

**Figure 10:**
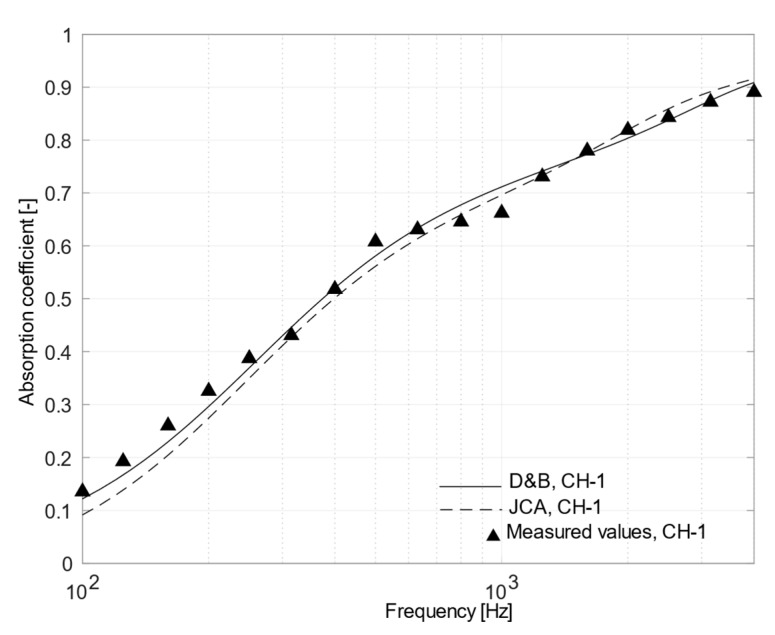
Plot of mean measured normal incidence absorption coefficients and those predicted using Delany and Bazley and Johnson–Champoux–Allard equation for CH-1 sample.

As a further confirmation of this behavior, Figure 10 shows the absorption curves referred to the sample CH-1, having the highest density and the highest air flow resistivity values. The absorption coefficients decreased significantly at high frequencies, reaching α = 0.73 at 1250 Hz. Such expected behavior was due to a higher flow resistivity value, higher than 60 kN∙s/m^4^. It is worth noting that D&B performed fairly well, despite the flow resistivity was, in this case, above the maximum recommended value.

As confirmed by Figure 9 and Figure 10, both phenomenological and empirical models estimated the acoustic behavior of the denser materials characterized by a higher air flow resistivity values (CH-1, CH-2, and GA-1) almost with the same good accuracy. On the contrary, the JCA model outperformed the D&B model in comparison with measured absorption coefficients of the less dense materials (CH-3, CH-4 and GA-2, GA-3) with lower air flow resistivity values. Likely, when flow resistivity increases, the two models tend to converge, because the tighter packing of the fibers makes resistivity play a major role also in the JCA model.

### 4.3. Effect of Air Gap

The effect of the air gap between the porous samples and the rigid backing was investigated by analyzing different placements of the sound absorbing materials under test. In view of what has been concluded in Section 4.3, theoretical models allowed a more accurate prediction of the experimental acoustic behavior of materials with a higher porosity, for which JCA model was more accurate than D&B. Thus, the more porous samples, CH-4 and GA-3, were tested by placing them in the impedance tube with 50, 100, and 150 mm air gaps and only the phenomenological model was used as comparison.

**Figure 11:**
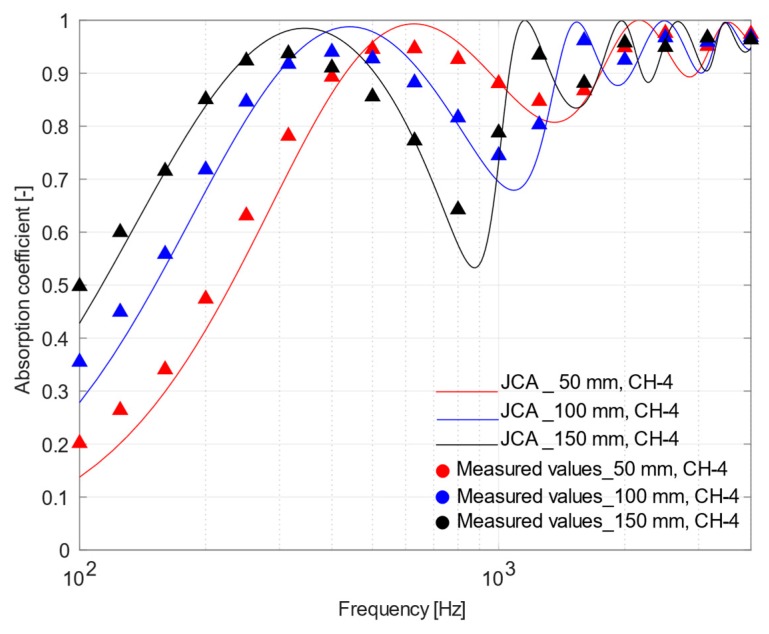
Plot of mean measured normal incidence absorption coefficients and those predicted using Johnson–Champoux–Allard equation for CH-4 sample placed at 50, 100, and 150 mm from the rigid surface.

**Figure 12:**
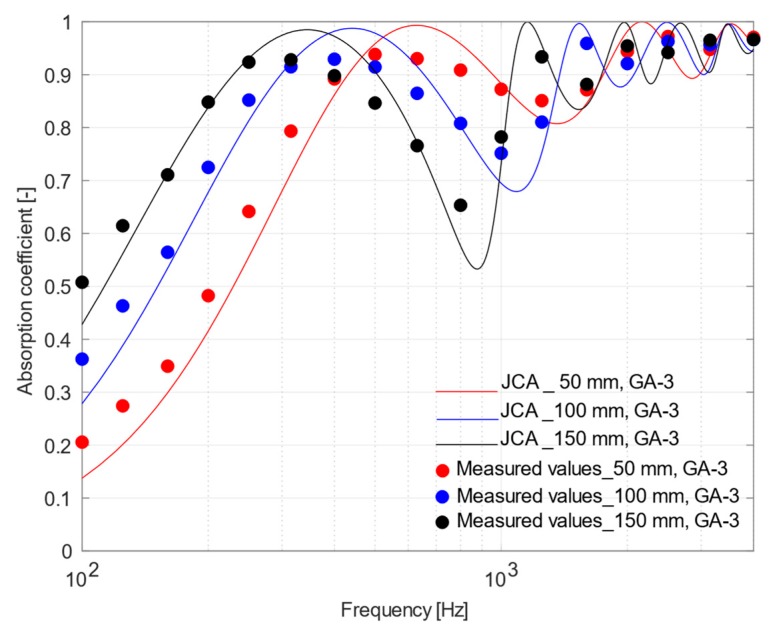
Plot of mean measured normal incidence absorption coefficients and those predicted using Johnson–Champoux–Allard equation for GA-3 sample placed at 50, 100, and 150 mm from the rigid surface.

It is well known that placing a porous material at a distance from a rigid surface is a valid technique to improve sound absorption at low frequencies, as an alternative to increasing the thickness of the material. In fact, Figure 11 and Figure 12 show that the peak value of the sound absorption curves measured for the two samples shifted towards lower frequencies as the air gap increased. The minimum, appearing at twice the frequency where the maximum is located, tends to be deeper when the air gap increases, but under diffuse field conditions a much smoother response is expected. Finally, it is worth noticing that, even under these conditions, the agreement between measured values and those predicted using the JCA model remains very good.

### 4.4. Ignitability Test

**Figure 13:**
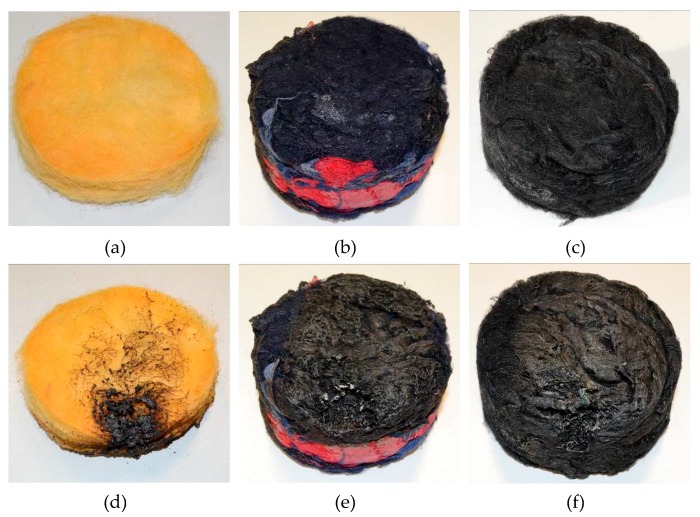
Samples used during ignitability test before (**a**–**c**) and after (**d**–**f**) flame application, (**a**,**d**) correspond to pressed wool fibers (no binders), (**b**,**e**) correspond to recycled wool with chitosan binder (CH-4), and (**c**,**f**) correspond to recycled wool fibers with gum Arabic binder (GA-3).

Results of the ignitability test are given in Figure 13 and show some interesting differences. It can be observed that pressed wool (Figure 13a,d) presented the best behavior, with a very limited flame propagation on the flat face (not exceeding 60 mm from ignition point), some smoke, and no droplets of melted material. Flame extinguished immediately after the burner was retracted. Samples with chitosan binder (Figure 13b,e) showed a quite different behavior, with a flame spreading up to the topmost part of the sample, limited smoke, and no droplets. The flame extinguished as soon as the burner was retracted, but very small carbonized portions kept burning for about 5 s, with smoke production. Finally, samples with gum Arabic binder showed a limited flame spreading on the flat surface (not exceeding 80 mm from the ignition point), copious smoking, and no droplets. No flaming and smoking was observed after the burner was retracted. Thus, the test pointed out significant differences that are worth being further investigated, but results are promising, also in the light of potentially improving this behavior by means of additives mixed to the binder [48]. 

## 5. Practical Implications and Limitations

The scope of the present paper was that of investigating the performance of composite materials obtained from the use of fibers derived from recycled textile wastes (100% merino wool), combined with different binders to be used as building materials with sound absorbing and thermal insulating properties. Results that were presented in the previous section confirmed that such materials have similar or better performance than conventional thermal insulating and sound absorbing materials. Thus, in order to understand whether they might be actually competitive, worth being used in the real world, and hence being industrialized in some way, it is important to answer to some questions: 

Do they provide comparable resistance to aging than conventional products? Do they provide comparable fireproof ratings as conventional materials? Additionally, above all, is their environmental impact lower, or equal, than conventional materials? 

Clearly, providing a specific answer to all of these questions would require further studies which are under way. However, it is possible to make some preliminary considerations based on literature evidences. With reference to aging and resistance to external attacks, wool is known to have a very good capacity for storing water vapor, thus preventing the material from getting damp. Consequently, wool naturally offers good resistance to mold and fungi. In addition, chitosan has been proved to contribute to further prevent mold and bacteria formation [49], and similar antibacterial effects have been shown by gum Arabic [50]. 

In terms of fire resistance wool is known to outperform any other textile fiber (both natural and synthetic), because it has a very high ignition temperature of 570–600 °C combined with a high limiting oxygen index (that measures the amount of oxygen needed to sustain combustion) and low combustion heat. Thus, in the light of the above features it is self-extinguishing. In addition, wool does not melt and fibers swell when heated, creating a tighter layer that prevents flame from spreading. However, as demonstrated by ignition tests, binders affected negatively such performance, particularly in terms of flame spreading (for chitosan) and smoke generation (for gum Arabic). Therefore, it becomes of primary importance to investigate either the chemical processes behind combustion and the use of alternative binders or flame retardant treatments. 

In terms of environmental impact of the binders, the figures that were presented in the introduction might be properly “rescaled” to take into account the actual amounts of materials that are required, based on the sample composition. Therefore, as the binding solution includes 15 g chitosan per kg water, and that the amount of binder is 60% the mass of the sample, this results in just 9 g of chitosan per kg of (wet) sample prepared. Thus, the higher environmental costs of chitosan production are only marginally reflected on the panel preparation. With reference to gum Arabic the situation is a bit different as the amount of dry product requested to manufacture 1 kg of composite panel is 120 g, which is much higher than chitosan. However, in the light of the positive effects related to Acacia tree cultivation, we might expect a lower overall impact. 

## 6. Conclusions

The experimental analysis of the thermal properties showed that all the samples have thermal conductivity ranging between 0.049 and 0.060 W/(m K), independent of the binder used. As expected, λ values linearly increased with increasing materials density and decreasing material porosity. 

Porosity variation also affected the air flow resistivity of samples, influencing their acoustic performance. It was observed that more porous samples were characterized by a lower air flow resistivity, showing a better sound absorption in the mid and high frequency ranges (with α higher than 0.8 at frequencies above 500 Hz). On the contrary, the increased air flow resistivity values of the less porous samples improved sound absorption at low frequencies yielding α as high as 0.5 from 315 Hz on. 

A comparison between measured and predicted absorption coefficients was useful to identify the Johnson, Champoux, and Allard model as the most suitable to perform any optimization exercise. In addition, using some of the selected samples in combination with air gaps allowed achieving α higher than 0.8 at frequencies above 315 Hz, using only a 5 cm gap. 

Further investigations are under way in order to define the mechanical characteristics of the samples and the practical application of the proposed solutions in the building industry. In addition, given the substantial equivalence between chitosan and gum Arabic in terms of performance, a life cycle assessment will be carried out to better clarify which solution is more eco-friendly.

## Figures and Tables

**Table 1:** Mixing ratio of binding solutions.

Solution	Solute [g]	Water [g]	Acetic Acid [g]
Chitosan	15	1000	5
Gum Arabic	200	1000	-

**Table 2:** Sample ID, bulk density (with uncertainty given in brackets), and fractional composition for wet and dry samples (in mass).

Sample ID	Bulk Density ρ	Fibrous Matrix	Binder	Fibrous Matrix	Binder
	[kg/m^3^]	[wet %]	[wet %]	[dry %]	[dry %]
CH-1	197(1.7)	40	60	95	5
CH-2	145(1.6)	40	60	95	5
CH-3	122(1.2)	40	60	95	5
CH-4	80(1.0)	40	60	95	5
GA-1	177(2.9)	40	60	78	22
GA-2	143(1.6)	40	60	78	22
GA-3	93(1.1)	40	60	78	22

**Table 3:** Summary of non-acoustic parameters for each sample.

Sample Code	Bulk Density, *ρ*	Porosity, *N*	Measured Tortuosity, *τ*	Estimated Tortuosity, *τ_est_*	Air Flow Resistivity, *σ*	Thermal Conductivity, *λ*
	[kg/m^3^]	[-]	[-]	[-]	[kN·s/m^4^]	[W/(m·K)]
CH-1	197(1.7)	0.86(0.002)	1.23(0.07)	1.08(0.0028)	66.3(7.8)	0.060(0.0005)
CH-2	145(1.6)	0.89(0.001)	1.11(0.05)	1.06(0.0008)	46.0(9.1)	0.055(0.0007)
CH-3	122(1.2)	0.91(0.001)	1.12(0.05)	1.05(0.0013)	16.5(2.7)	0.052(0.0006)
CH-4	80(1.0)	0.94(0.001)	1.20(0.18)	1.03(0.0016)	11.7(2.2)	0.049(0.0005)
GA-1	177(2.9)	0.87(0.0014)	-	1.08(0.0019)	44.7(5.0)	0.059(0.0012)
GA-2	143(1.6)	0.90(0.0003)	-	1.06(0.0004)	23.6(2.5)	0.056(0.0006)
GA-3	93(1.1)	0.93(0.0004)	-	1.04(0.0006)	14.4(2.3)	0.050(0.0005)

**Table 4:** Coefficients used to feed the Johnson–Champoux–Allard (JCA) model.

Sample Code	Shape Cactor, s	Ratio, Λ′/Λ	Mean Absolute Error
	[-]	[-]	[-]
CH-1	2.0	2	0.0172
CH-2	0.8	2	0.0240
CH-3	3.0	2	0.0295
CH-4	2.0	2	0.0334
GA-1	1.5	2	0.0307
GA-2	2.5	2	0.0304
GA-3	2.0	2	0.0263
